# Data-driven models and digital twins for sustainable combustion technologies

**DOI:** 10.1016/j.isci.2024.109349

**Published:** 2024-02-27

**Authors:** Alessandro Parente, Nedunchezhian Swaminathan

**Affiliations:** 1 Aero-Thermo-Mechanics Department, École polytechnique de Bruxelles, Université libre de Bruxelles, Avenue Franklin D. Roosevelt 50, 1050 Brussels, Belgium; 2 WEL Research Institute, Avenue Pasteur 6, 1300 Wavre, Belgium; 3 Brussels Institute for Thermal-fluid systems and clean Energy (BRITE), Université libre de Bruxelles and Vrije Universiteit Brussel, 1050 Ixelles, Belgium; 4 Department of Engineering, Hopkinson Laboratory, Cambridge University, Cambridge CB2 1PZ, UK

**Keywords:** Machine learning, Energy sustainability

## Abstract

We highlight the critical role of data in developing sustainable combustion technologies for industries requiring high-density and localized energy sources. Combustion systems are complex and difficult to predict, and high-fidelity simulations are out of reach for practical systems because of computational cost. Data-driven approaches and artificial intelligence offer promising solutions, enabling renewable synthetic fuels to meet decarbonization goals. We discuss open challenges associated with the availability and fidelity of data, physics-based numerical simulations, and machine learning, focusing on developing digital twins capable of mirroring the behavior of industrial combustion systems and continuously updating based on newly available information.

## Introduction

The race for carbon neutrality by 2050 is underway.^1^ The current paradigm of considering the energy consumption in various sectors such as transport, industry, domestic application, etc. individually will impede reaching climate neutrality.^1^ A holistic view of an interconnected system linking different sectors with appropriate energy carriers and shared infrastructures needs to be developed. The core of such an approach is energy efficiency and electrification, which relies massively on renewable energies.^2^ Whether this is feasible or not is a complex question with significant uncertainties.^3^

About 50% of energy needs in hard-to-abate industries can be met through electrification.^4^ They pose significant challenges for reducing their carbon emissions. Heavy industries such as steel, cement, and aluminum rely on high-temperature heat (e.g., >1,300 K)^5^ that currently requires combustion to be cost-effective. In addition, producing some chemicals and fertilizers, such as ammonia, relies on non-energy sources (i.e., natural gas) as feedstock and emits greenhouse gases. The complexity and integration of these industrial processes make it difficult to find carbon-free alternatives, and the significant investments for these alternatives can challenge their implementation for industries. In sectors such as heavy-duty transportation, electrification is not straightforward because of the requirement of high-density, localized energy sources that fuels can only provide. For example, aviation, shipping, and trucking require fuels that cannot be substituted with solar panels or batteries due to the high energy density required. The World Energy Outlook from International Energy Agency (IEA)^6^ recently noted, “… there are end uses for which it is not feasible or cost-effective to electrify directly with the technologies currently available on the market or likely to be commercialized in coming decades. Around half of energy demand in energy-intensive industries is for high grade process heat (above 400°C), which is challenging to electrify with current technologies. Ships, planes, and heavy trucks make up around 85% of transport non-electricity demand in 2050; at present it is very difficult to see how most of the energy they use could be electrified,” stressing this criticality.

Hard-to-abate sectors with challenges for electrification will need to implement a series of measures to meet the ambitious decarbonization goals. Top priorities^7^ are represented by energy efficiency and demand side management, electrification of heat and processes up to the maximum achievable levels, carbon capture and utilization, and the use of carbon-neutral fuels, renewable synthetic fuels, or biofuels. In this context, hydrogen and hydrogen carriers like ammonia enjoy renewed and rapidly growing attention as the hard-to-abate industries emit nearly 30% of the global CO_2_.^6^ Energy-intensive industries are exploring replacing fossil fuels with synthetic and renewable energy carriers entirely or in combination with conventional fuels (natural gas) and hybrid configurations involving other types of heating. Many fundamental questions and new challenges must be addressed while developing the critical approaches to achieve such technological advancements. Specifically, the impact of changing fuel/burner technology on the combustion processes in large industrial furnaces, the associated pollutants emission, the overall process efficiency, and the effects on the product characteristics still need to be fully understood. This knowledge is critical for implementing major technological changes to large industrial assets, for which experimental testing is neither comprehensive nor feasible at the scales of interest. Also, current computational tools do not offer the required fidelity and computational efficiency to evaluate potential scenarios to be explored for decarbonizing these industries.

**Figure 1:**
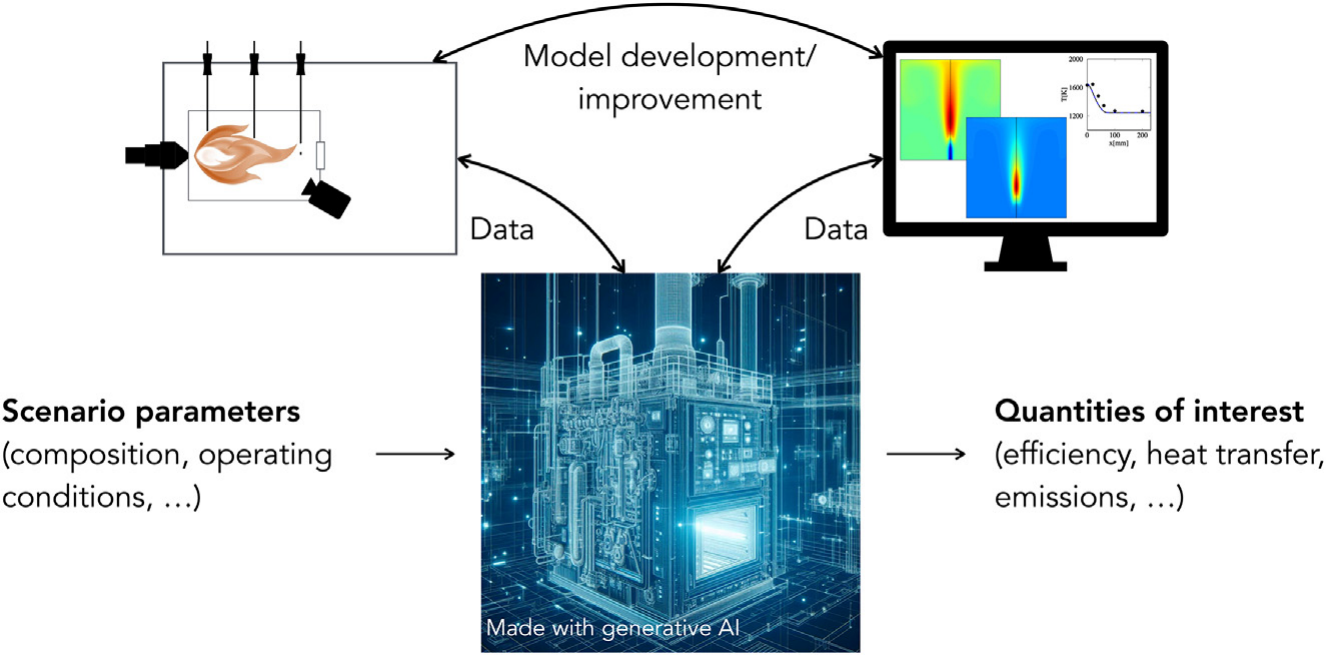
A schematic of a digital twin (DT) with data flow: cyber-physical infrastructure can drive decarbonization in hard-to-abate industries creating a link between physical assets and virtual models to assess reliably and affordably the impact of renewable synthetic fuels on the combustion processes, the associated pollutants emission, the overall process efficiency, and the effect on the characteristics of the products

This perspective paper discusses the key developments in combustion science and its hybridization with other cutting-edge research topics required to develop a pervasive, physics-aware, data-intensive framework enabling sustainable combustion technologies and using renewable fuels for energy-intensive applications. The challenges and enabling technologies associated with deploying digital twins (DTs) for various stakeholders have been discussed by Rasheed et al.^8^ This paper highlights some specific challenges associated with energy-intensive industries and key requirements from a modeling perspective. We focus on the opportunities offered by DTs and cyber-physical infrastructures by incorporating heterogeneous (both in type and accuracy) data streams into predictive models with well-defined confidence, highlighting the challenges and open research needs (Figure 1).

## The role of data in the development of sustainable combustion technologies

Combustion systems involve wide-ranging time and length scales, transitions, bifurcations, and multiple interacting physical phenomena that are highly complex to predict. While using turbulent combustion models has become common across industries, their current predictive capabilities do not yet meet the required standards for new design and regulation,^9^ especially regarding pollutants and operational stability limits.^10^^,^^11^ The advent of exascale computing has potentially unlocked the application of direct numerical simulations (DNSs) of combustion systems limited beyond specific aspects of turbulent combustion physics and building blocks. Still, not all relevant dimensionless numbers/conditions are numerically accessible. Moreover, exascale facilities are only available to a handful of researchers, and their availability for the industry and routine simulations is miles away.

DNSs produce vast data in some dimensions (spatial and temporal resolution) but very sparse in others (flow conditions, fuel composition, etc., to name a few); hence, generalizing the knowledge gained becomes challenging. Still, DNS data have rich information that can help decoding complex turbulence-chemistry interactions and guide the development of filtered and low-fidelity modeling approaches for quicker evaluations.^12^^,^^13^^,^^14^ Indeed, combustion physics and its interaction with turbulence are small-scale phenomena that are expected to be quite universal, and, hence, the ML-enhanced models developed for these complex small-scale physics are expected to hold well across a wide range of conditions and problems.

Some recent attempts have been made to create databases and benchmark cases (e.g., the BLASTNet^15^). A recent review showed that more than 200 DNS studies on turbulent reacting flows over the last two decades were published during the last two decades.^16^ However, very little of these data have become available to the broad combustion community. Challenges to be overcome are primarily related to standardization, both concerning the shared information and the codes used to generate the data. Efforts such as BLASTNet are great initiatives to define minimal requirements for datasets in terms of (meta)data required to associate them to a specific code and recreate specific labels for machine learning (ML) training. Handling code variability represents a different challenge and would require the inclusion of metrics to assess the quality of data. Finally, the typical size of DNS combustion data can impede data sharing, especially for combustion cases. This can be addressed by creating one or more centralized and maintained data storage facilities with easy access by any community member, ensuring that the data can be identified and searchable.

Similarly, high-fidelity measurements are available only for laboratory-scale systems, and the amount and accuracy of the measurements decrease going from small to large scales. Realistic combustion environments are typically very harsh, and sophisticated combustion diagnostics can hardly withstand them, limiting the diagnostic options and excluding the use of laser diagnostic techniques in most applications. Moreover, even with the most advanced diagnostic techniques, only a few variables of interest are accessible in industrial combustion systems, with limited spatial and temporal resolution. Differently to DNS data, the sharing practices for combustion experiments are well consolidated in the combustion community, and several workshops actively define benchmark cases for the simulation of different combustion systems.^17^ Velocity and scalar measurements are provided for the validation of numerical simulation approaches. However, while there is a tendency to define benchmark cases of increasing complexity and closer to applications of practical interests, available data remain fundamentally limited to laboratory-scale equipment. Recently, efforts have been made toward developing scale-bridging systems to fill the gap between laboratory units and real applications, particularly in the field of advanced combustion technologies enabling the combustion of renewable synthetic fuels.^18^^,^^19^^,^^20^^,^^21^ While vital, these initiatives still require further development, especially concerning the standardization of the shared data and their attributes.

Designing a complex system is an iterative process requiring many model evaluations and extensive experimental testing. As such, it cannot rely solely on high-fidelity tools, admitting they are available, nor can it be carried out directly at the scale of interest. Indeed, neither experimental testing nor numerical simulations are self-sufficient, and a synergistic combination is required. A combination of approaches is required to bridge information from high- and low-fidelity models across scales and data types. Data-driven approaches and artificial intelligence have tremendous potential to enable a step change in combustion science and technology. A synergetic combination of experiments, simulations, and data-driven methods offers huge potential to address challenges of calibrating low-fidelity models, their parameterization, and the integration of heterogeneous data streams at different scales in calibration and model update processes. Specifically, research efforts to combine ML with physics-based knowledge are promising^22^ to enable the pervasive use of data-driven methods and develop reliable and effective reduced-order models (ROMs) for capturing the underlying hidden relationship between inputs and quantities of interest.

## DTs of industrial combustion systems: Where do we stand?

Combining simulations and measurements is key to developing DTs of large-scale combustion systems that can predict combustion evolution in real time and act as soft sensors. Indeed, extensive use of physical sensors in harsh environments like furnaces is infeasible, and deploying laser metrology for industrial combustion systems is difficult. Moreover, most sensors provide single-point information on a single parameter so that only a tiny portion of the system and a small set of physical/chemical parameters can be monitored. Gartner named DTs as one of the Top 10 Strategic Tech Trends 2018,^23^ and they are now mainstream and considered an integral part of the Fourth Industrial Revolution. The quick adoption of DTs is mainly connected to the faster spread of enabling technologies: the cloud and internet of things (IoT).

Among many definitions, one can describe a DT as *an integrated multi-physics, multi-scale, probabilistic simulation of an as-built system enabled by a digital thread that uses the best available models, sensor information, and input data to mirror and predict activities/performance over the life of its corresponding physical twin*.^24^^,^^25^^,^^26^ There are five components of a DT,^27^ viz., (i) the ability to simulate the system functions over its life cycle, (ii) synchronizing the digital and physical systems, (iii) real-time data integration, (iv) modeling the entire physical state space, and (v) its service functionalities. Achieving these five components for industrial combustion systems is very challenging. These systems involve turbulence and combustion, which are still unsolved problems in classical physics and chemistry.^9^ Their interactions take the challenges to a higher level, depending on the overlap between chemical and turbulent scales, resulting in many combustion regimes with fundamentally different reacting structures, morphologies, and topologies.^3^^,^^10^

Black-box approaches have been widely used in combustion to create static input-output maps^28^ and system identification^29^ to predict macroscopic quantities such as exhaust gas emissions and temperature and to detect oscillatory patterns such as thermoacoustic instabilities, respectively. In combustion, neural networks have been used to evaluate and tabulate reaction rates,^30^^,^^31^^,^^32^^,^^33^ as well as to estimate modeling errors in large eddy simulations (LESs).^12^^,^^34^ Black-box approaches can be very powerful, although not equipped with the guarantees of physics-based approaches. To cope with that, non-linear system identification techniques were proposed to enlighten black-box approaches by discovering the underlying physics.^35^

An interesting approach to generate combustion ROMs is the use of chemical reactor networks (CRNs),^36^^,^^37^^,^^38^^,^^39^^,^^40^^,^^41^^,^^42^^,^^43^^,^^44^ which fall in the category of gray-box models, as they combine a theoretical structure, i.e., the canonical reactors, with data to generate the network. The approach can also be regarded as an example of multi-fidelity methods, being high-fidelity tools (i.e., computational fluid dynamics, CFD) used to construct the reactor network and the simplified network model used to evaluate pollutant emissions and other quantities, using detailed chemical mechanisms. These techniques are particularly useful for large and complex systems, such as furnaces or gas turbines. However, their current overall fidelity and generalizability are limited by the high-fidelity simulations required to generate the network structure, indicating an interest in data-driven approaches in this area of research.^45^

ROMs based on projection methods (such as principal-component analysis [PCA] and other techniques) have been employed for experimental and numerical combustion data.^46^^,^^47^^,^^48^^,^^49^^,^^50^^,^^51^^,^^52^^,^^53^^,^^54^^,^^55^ The direct evolution of the modal coefficients in CFD codes (i.e., intrusive ROMs) has been quite limited for non-linear problems like combustion due to the high cost of the associated Galerkin projection and the complexity of coupling them to legacy and commercial CFD codes.^56^ The application of non-intrusive projection-based ROMs has been, on the other hand, quite successful in combination with non-linear regression approaches such as Gaussian process regression (GPR),^57^ neural networks, and polynomial chaos expansion (PCE).^58^ Three-dimensional CFD simulations were combined with sensor data to develop the DT^59^^,^^60^ of a semi-industrial furnace operating with methane-hydrogen mixtures and full hydrogen charges. The DT could predict scalar fields and, integral and point quantities with very high accuracy.

Other attempts at developing DTs of industrial applications involve the development of a simplified physics-based model with a set of coefficients tuned with an optimization method,^61^ building ROMs for specific variables of interest (e.g., emissions, vibrational data, …), and building estimators via regression algorithms for the system health states.^62^^,^^63^

## DTs of industrial combustion systems: What do we need to move forward?

The current state of the art highlights five key points for unlocking DT potentials for energy-intensive industries: the training data, data analysis and feature extraction, adaptive simulation frameworks and subgrid models, ROMs, and DTs with lifelong learning capabilities. Although these aspects are discussed for turbulent combustion systems, they are relevant for closely related multi-physics flow problems encountered in power generation, process industries, orbital re-entry, air pollution and atmospheric flows, and biological flows, to name a few.

### Training data

Collecting representative, accurate, and reliable training data is central for any data-driven approach. These datasets must cover a wide range of operating conditions and be representative of different potential applications. When looking at fields where ML prospers, such as advertising, image, or speech recognition, one realizes that the impact of each ML prediction is small and adds up to many similar decisions for which the training data are so vast that virtually all tasks may be regarded as interpolation. The situation for turbulent flows is very different. Indeed, the accuracy and physical realizability of reacting flow simulations are mandatory, thus requiring interpretable, explainable, and generalizable ML algorithms.^64^

Experimental data are required to validate models; they are our ultimate window into reality. However, data availability at the industrial scale is scarce due to the challenges of performing full-scale, high-fidelity experiments.^11^ Configurations bridging the laboratory and industrial scales are required to acquire data whose quantity and quality are relevant to the application of interest. The concept of scale-bridging data is paramount for DTs of industrial systems and represents a key area of research. The notion of scale-bridging data is not new and can be related to the validation hierarchies introduced by Oberkampf and Trucano.^65^ The challenge lies in identifying systems with a degree of physical interactions relevant to the final application of interest but allowing isolating and controlling quantities of interest.

Numerical simulations can offer a level of detail that is yet unattainable in experiments since physical sensors cannot be massively deployed because of harsh conditions and costs. Moreover, data must cover a wide range of operating conditions and be representative of different potential applications. High-fidelity simulations of turbulent reacting flows are still limited to small domains and archetypal configurations due to their complexity and cost, both computational and monetary. Moreover, they generate vast data in some dimensions (thanks to the fine spatial and temporal resolutions). Still, they are very sparse in others (flow conditions and fuel composition), posing challenges for generalization.

Ideally, one would rely on DNS data, but this is far from a viable solution. Massive datasets are available for very few operating conditions (regarding chemical composition, turbulence level, turbulence/chemistry interactions, etc.). The impact of each evaluation on the results is critical, and guarantees on performances and uncertainty in the predictions must be provided. This points out that one must be cautious while extending ML approaches from one case to another since these approaches are also prone to overfitting.

Generating an appropriate dataset for the optimal representation of a system is still an open question, not only for chemically reacting flows.^66^^,^^67^^,^^68^ In tabulated chemistry approaches, it is common practice to rely on simple chemistry-based simulation attempting to replicate the ensemble of state spaces encountered in an actual three-dimensional numerical simulation.^9^ This requires prior knowledge of the system since the system is constrained on a pre-computed manifold. Moreover, challenges can arise when multiple combustion regimes coexist, indicating that the state-space trajectories for one archetypical flame are insufficient. Merging datasets from different archetypical flames is also attempted using *ad hoc* and case-specific approximations.

The opportunities offered by ML are extremely promising, such as developing unsupervised methodologies to “nest” different reactor and flame types into a single database for training and developing ROMs and tailoring modeling to specific local regions in numerical simulations.

### Data analysis and feature extraction

Experiments and high-fidelity simulations of combustion typically generate massive amounts of data. Hence, big data in combustion have been a reality for almost three decades.^69^ This is also aided by the advent of petascale and exascale computing which allows DNS for close-to-reality conditions. These big data are typically used for model development and validation.^12^^,^^13^^,^^14^ Many data-processing techniques for combustion have been developed in the past 50 years, and these techniques can be used for both physical and numerical experiments. While these analyses have strongly relied on domain expertise and heuristic algorithms, they can certainly be regarded as an early application of ML.

A key point is to link unsupervised feature extraction and domain knowledge. Reaction progress variable is, for instance, a key central concept in combustion theory and modeling. Nevertheless, no consensus exists in the literature about its definition and generalization.^70^ Recent studies based on modal decomposition techniques such as PCA^50^^,^^67^^,^^71^^,^^72^ have suggested that PCs can be associated with distinctive flame processes, linking feature extraction algorithms and domain knowledge. Other challenges in the analysis of combustion datasets are related to the representativity and sample density of different regions of the state space,^73^ the state-space pre-processing,^53^^,^^72^^,^^74^^,^^75^^,^^76^ the set of state variables to be selected^54^^,^^72^^,^^76^^,^^77^^,^^78^^,^^79^^,^^80^^,^^81^^,^^82^ and the associated impact on the topology,^83^^,^^84^ parameterization,^76^ and (supervised) regression of the low-dimensional manifold.^72^^,^^82^ To date, solutions to this problem are scarce and case specific, despite the significant potential impact on the performances of predictive combustion simulations.

A quantitative manifold-informed method to assess the manifold topology has been proposed,^85^ opening the way to an optimal (from the topological perspective) parameterization of the low-dimensional manifold using both linear (e.g., using PCA) and non-linear (e.g., autoencoders) projections, and the adaptive mapping/regression of the variables of interest accounting for local manifold characteristics. Using linear/non-linear activation functions (for the encoding and decoding processes, respectively) combined with topology-aware loss functions is particularly appealing. Indeed, optimized manifold topologies can be achieved by leveraging the effect of the back-propagation algorithm on the network weights while keeping a linear projection that has proven useful to derive physics-based transport equations of reacting scalars in the principal-component space.^86^

### Adaptive simulation frameworks and subgrid models

**Figure 2:**
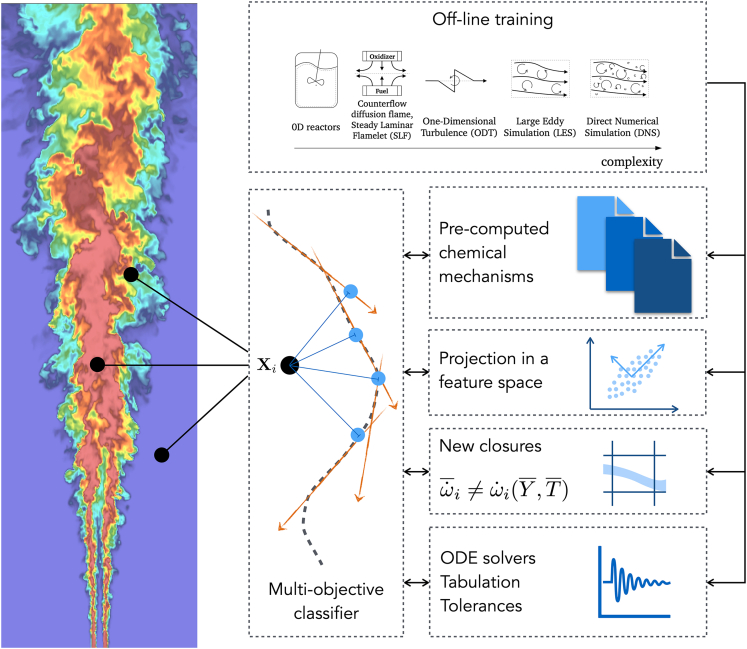
Adaptive simulation framework On-the-fly classification for dynamic, turbulent combustion simulations, adapting the chemical mechanism, the number of chemical species, the combustion closure, and the numerical settings to the local flow conditions.

The development of simulation-based ROMs or DTs presents several challenges related to the computational power required to generate enough numerical simulations of different fidelity. Considering the size of detailed chemical mechanisms and the typical dimensions of combustion systems involved in energy-intensive industries, it is crucial to develop computational approaches aiming for local comprehensiveness. In connection with the discussion on ***training data***, unsupervised clustering approaches appear particularly promising to open the way for fully adaptive models that adjust the chemical mechanism, the number of transported species, the closures, and the numerical solvers and tolerances in CFD simulations, depending on the local flow conditions. The coupling of clustering with dimensionality reduction using PCA has proven particularly effective in identifying regions of the state space with structurally different characteristics and distinct physical processes and in developing adaptive chemistry models.^87^^,^^88^ This concept has been demonstrated for adapting the complexity of the chemical mechanism locally in numerical simulations but can be extended to the entire simulation workflow shown in Figure 2, with applications beyond the sole combustion field. Subfilter modeling for high-fidelity LES is an appealing area for data-driven models. In combustion problems, temperature fluctuations can be as high as several hundred Kelvins. Since Arrhenius reaction rates are highly non-linear functions of temperature, accurate statistical closures for filtered approaches cannot be based on an expansion around mean properties.^9^ The task of a combustion model is to describe the unresolved scales based on the information available during a simulation. Data-driven methods significantly impact the design of improved subgrid combustion models.^89^^,^^90^^,^^91^^,^^92^^,^^93^^,^^94^^,^^95^^,^^96^^,^^97^^,^^98^ While replacing the chemical source term with black-box approaches may be helpful for recurring numerical simulations, such an approach is limited in scope because of its intrinsic inability to generalize. More interesting options include embedding ML in physical models or incorporating physical knowledge into ML models.

An example of the first approach is to define data-driven correction terms in the form of summative or multiplicative residuals (i.e., using ML for correction terms while preserving a physics-based approach) in the framework of different closure paradigms, flamelet-like to PDF-like approaches, from conditional closures to reactor-based approaches.^99^^,^^100^ Incorporating physical knowledge into ML algorithms can be achieved by promoting the compliance of the results with the known laws of physics for the problem at hand. In neural networks, this can be accomplished by tailoring the loss function to include terms penalizing the violation of specific conservation principles or the entire residual of a governing equation.^101^ In combustion, examples involve physics-informed neural networks applied to stiff chemical kinetics problems^102^ and physics-informed enhanced super-resolution generative adversarial networks (PIERSGANs) for subfilter modeling.^103^

A final important remark concerns the actual use of ML-enhanced closures in turbulent combustion simulations. Many of the proposed approaches have been only demonstrated in *a priori* testing, but only a few have made it to *a posteriori* simulation.^103^ Models showing good performances in an *a priori* framework could ultimately fail in an actual simulation, particularly those not accounting for the problem physics in any form, resulting in unphysical predictions and uncontrolled error propagation. Finally, there are challenges in posterior validation related to the numerical implementation and the coupling with the CFD codes, which are not directly relevant to the focus of this paper.

### ROMs

**Figure 3:**
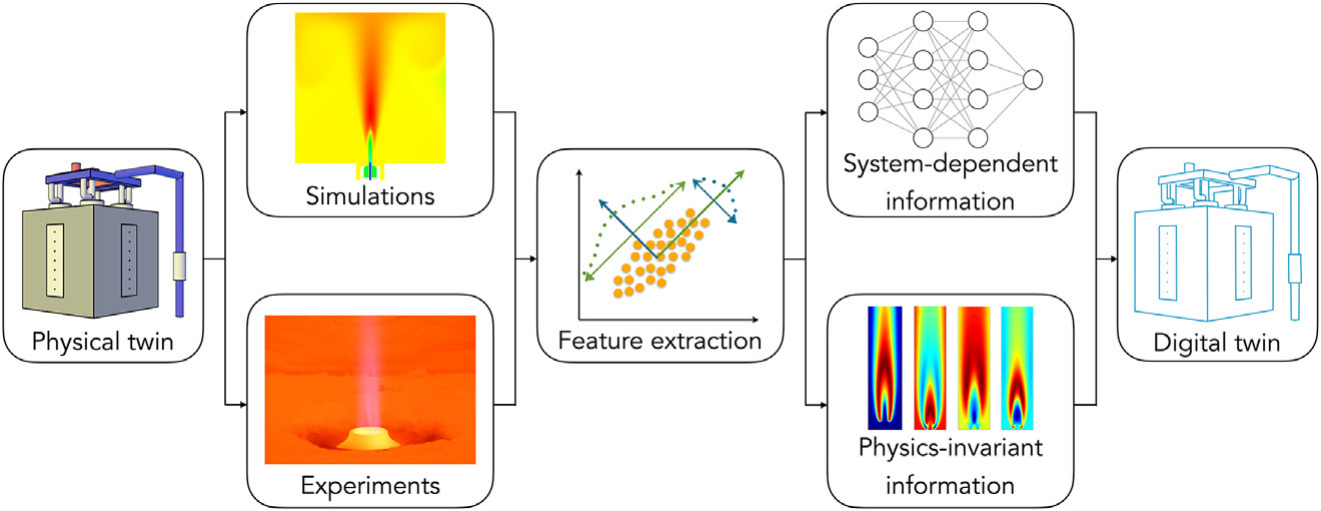
A digital twins development framework based on heterogeneous data streams, feature extraction/dimensionality reduction, and ML algorithms

Complex systems design is an iterative process requiring several model evaluations and answers for “what if” scenarios. As such, it cannot rely solely on high-fidelity tools (such as CFD simulations). A combination of approaches is needed to bridge information from high- and low-fidelity models into a reduced representation of a complex system. Engineers have traditionally collected the results of experiments and numerical simulations through correlations to design and optimize systems. Today, the challenge is developing strategies to assimilate data of different natures and fidelity into a reduced representation of the system of interest. Approaches based on the combination of dimensionality reduction (using linear techniques such as PCA) and non-linear regression have been successful^59^^,^^60^ in developing simulation-based DTs able to mimic the behavior of complex three-dimensional CFD numerical simulations (Figure 3). To further reduce prediction errors in the design space, non-linear dimensionality reduction (e.g., autoencoders-like) appear particularly well suited. Indeed, classic PCA tends to overestimate dimensionality, prompting the exploration of non-linear methods like kernel PCA,^54^^,^^73^ T-distributed stochastic neighbor embedding (T-SNE),^104^ and autoencoders.^105^ While non-linear methods enhance dimensionality reduction, they are more challenging regarding physical interpretation and can be computationally costly since they involve numerous hyper-parameters to calibrate.^106^

Associating a level of confidence/uncertainty to the predictions also appears to be of paramount importance for ROMs, prompting the use of techniques such as GPR and Bayesian networks.

Finally, strategies need to be explored for the efficient fusion of data of different fidelity. The characterization of an industrial furnace may be, for instance, unfeasible, using LESs only. A common strategy in computer-aided design is to perform a decreasing number of simulations of increasing complexity: operating conditions are generally explored using lumped systems (e.g., 0D reactors) and one-dimensional models. Systems’ performances are then broadly investigated using reactor networks and RANS simulations, and few conditions of interest are analyzed in detail using LES. Moreover, when performing RANS or LES simulations, the computational effort can strongly vary depending on the level of fidelity employed to describe chemical kinetics (chemical schemes for methane combustion can, for instance, range from ∼20 to more than 100 species), the number of chemical species impacting the computational cost more than quadratically.^9^ There is an opportunity to exploit all the available data to generate and refine ROMs of complex combustion systems using heterogeneous data. Several multi-fidelity models, such as non-intrusive polynomial chaos,^107^ CoKriging,^108^ and radial basis function (RBF),^109^ have been proposed in the literature. Techniques seeking to align the latent spaces spanned by the predictions of different fidelities^68^ have been successfully applied in aerodynamics and other fields, demonstrating reduced computational costs and improved accuracy. Fusing low-fidelity and high-fidelity data in multi-fidelity ROM balances accuracy with computational budget constraints. This involves replacing some high-fidelity simulations with low-fidelity ones to minimize training costs while accepting a limited accuracy loss in the resulting ROM. The fundamental question is how many high-fidelity simulations can be replaced with low-fidelity ones in each design of experiment to train a ROM with satisfactory prediction accuracy.

### DTs with lifelong learning capabilities

**Figure 4:**
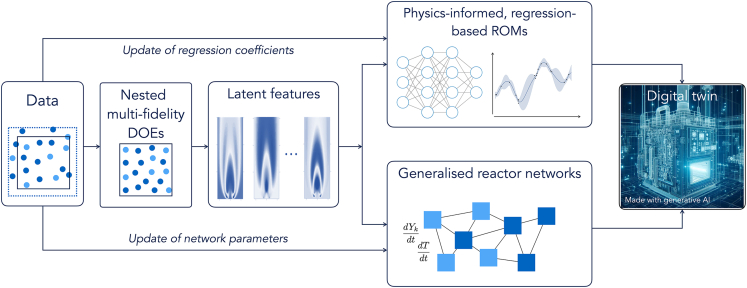
Schematic of the digital twin generation/adaptation process DTs can be updated using new data to modify the mapping between the feature and state spaces and between the input conditions and reactor network parameters. A new training process shall be carried out for new data significantly exceeding the original DOE.

Once built, ROMs can be used to replicate the numerical simulations. As such, they are affected not only by the error intrinsic in the ROM generation process but also by the limitations of the first-principle model (uncertainty in boundary conditions model parameters, numerical and experimental errors, …). Strategies to update the DT (Figure 4) once in operation (lifelong learning) can profit from the vast literature in the field of data assimilation (DA) techniques. The latter have gained momentum in the combustion community to update numerical simulations based on high-fidelity measurements,^110^ to calibrate physics-based ROMs,^109^^,^^111^ and to update DTs based on few experimental measurements.^112^ ROMs based on dimensionality reduction and regression methods are particularly appealing in this framework for the possibility of updating the regression coefficients (of a GPR regression or a neural network) based on the available data stream. ROMs based on CRNs can also be very appealing for their gray-box nature: data-driven algorithms can be designed to develop generalized CRNs consisting of combinations of ideal reactors for which the governing equations are known. The chemical network can be generated starting from simplified CFD simulations (thus relying on the multi-fidelity concept highlighted above) using unsupervised clustering algorithms modified to enforce the determination of spatially continuous clusters (using geometrical penalties or graph algorithms) to ensure spatial continuity of the identified clusters. The availability of multiple CFD simulations can potentially allow extraction of different CRNs, each tailored to precise simulation settings. To overcome the case-specific nature of the approach, a mapping between input conditions in the design space and network parameters (reactor volumes and mass exchanged between reactors) can be established using non-linear regression techniques. This approach can relate changes in internal mixing, induced by differences in operating conditions, to an adaptive set of network parameters, thus delivering a generalized CRN model that can represent the system under a wide range of operating conditions.

## Conclusions and prospects

We emphasize the pivotal role of data in combustion technologies for energy-intensive industries. The inherent complexity of combustion systems necessitates innovative approaches, where data-driven methodologies and artificial intelligence emerge as transformative tools. The vision of creating robust and predictive DTs for large-scale combustion assets presents a promising avenue toward achieving sustainable combustion systems, enabling the potential of renewable synthetic fuels. However, some key challenges need to be addressed. These include obtaining representative training data, developing data analysis tools and adaptive simulation frameworks, creating accurate ROMs, and enabling DTs with lifelong learning capabilities. The need for a multi-disciplinary approach is highlighted to address these challenges and establish a comprehensive, physics-aware, data-intensive framework for sustainable combustion technologies in the realm of energy-intensive industries.

## Limitations of the study

This paper reflects the authors’ perspective on the role of data in advancing combustion technologies for energy-intensive industries. It reflects our interpretation and synthesis of the existing literature and trends and combines it with our vision of how the field will evolve. While we outline key challenges and potential solutions, the implementation and effectiveness of these strategies will vary depending on specific industrial contexts and technological advancements.
